# Tissue donation in forensic cases: a retrospective study from Sweden

**DOI:** 10.1007/s10561-026-10232-4

**Published:** 2026-07-08

**Authors:** Kajsa Kepplerus, Malin Pauli, Astrid Ohlin Åstrand, Jesper Greby, Ali-Reza Rezaie, Kristoffer Björkstrand, Brita Zilg, Nina Heldring

**Affiliations:** 1 Department of Forensic Medicine, Swedish National Board of Forensic Medicine, Sölvegatan 25, 223 62 Lund, Sweden; 2 Department of Forensic Psychiatry, Swedish National Board of Forensic Medicine, Alfred Nobels Allé 41, 141 52 Stockholm, Sweden; 3 Department of Clinical Neuroscience, Centre for Psychiatry Research, Karolinska Institutet, & Stockholm Health Care Services, Region Stockholm, Stockholm, Sweden; 4 Department of Forensic Medicine, Swedish National Board of Forensic Medicine, Blombäcks väg 5, 171 65 Stockholm, Sweden; 5 Department of Oncology-Pathology, Karolinska Institutet, 171 77 Stockholm, Sweden; 6 Department of Laboratory Medicine, Centre for Biomolecular and Cellular Medicine, Karolinska Institutet, 141 86 Stockholm, Sweden

**Keywords:** Donation feasibility, Manner of death, Donation registry, Consent, Tissue and organ procurement

## Abstract

Loss of a loved one under traumatic circumstances is challenging and often requires families to make difficult decisions. Forensic autopsy cases frequently involve sudden or traumatic deaths but do not preclude tissue donation or contact with next-of-kin. Many deceased individuals in these contexts are young and medically suitable for donation, making it important to understand when such donations are feasible to ultimately expand the pool of available tissues. This study investigates how next-of-kin interpret the deceased’s presumed donation wishes based on manner of death, age, and sex. Using registry data from the Swedish National Board of Forensic Medicine, we analyzed demographics and consent outcomes was used to assess factors associated with next-of-kin consent. Of 1159 donation assessments, 691 cases involved next-of-kin contact. In cases without prior official donor declaration (556 individuals), consent was about twice as likely after natural death compared with accidents (OR 2.42) or suicide (OR 1.73). Among children aged 0–17, next-of-kin were markedly more likely to interpret boys’ wishes as positive compared with girls (OR 7.59), while no sex differences were seen in adults. These findings show that tissue donation remains feasible in forensic cases and that both manner of death and age shape family interpretations, underscoring the need for tailored communication.

## Introduction

The loss of a loved one, particularly under traumatic circumstances, represents a profoundly challenging experience and often involves the need to make several difficult decisions. One such decision may concern the deceased individual’s stance on tissue donation. Although this information is sometimes documented, this is not always the case, and the individual’s preferences may also have changed since any prior registration in the donation registry. It is therefore of considerable public interest to ensure that tissue donation is conducted under appropriate ethical and procedural conditions and promoted to the greatest possible extent, in order to meet the demand for tissues for transplantation, research, and education. While the decision-making process can be stressful for the next-of-kin, the fact that the donation can help others can also be a comfort and help the grieving process (Ralph et al. 2014). Individuals in Sweden may from 15 years of age register their consent or non-consent to organ or tissue donation in a national donation registry. Stem cell or sperm/egg cell donation is covered by other registries. Individuals may revise or withdraw their registration at any time and the most recent entry is considered valid. If death occurs and no record in the national donor registry exist, the individual’s wishes are determined through consultation with next-of-kin. When preferences are unknown, presumed consent applies; however, relatives may oppose donation if they believe the individual would have declined. In Sweden, a forensic medical examination of a deceased individual may be conducted according to The Swedish Autopsy act (1995:832) when it is considered significant for investigating a death that occurred under circumstances suggesting a criminal act, suspected medical error or negligence or when death is believed to have resulted from external factors ( in order to determine the cause of death or to obtain information of particular relevance to environmental protection, occupational safety, traffic safety, or similar public interests) (SFS 1995). Additionally, a forensic examination may be performed when necessary to establish the identity of the deceased. Forensic autopsy cases often involve young, healthy individuals suitable for tissue donation. Up to 19% of all tissues (Malm et al. 2016) and 10% of corneal donations (Bofill-Rodenas et al. 2019) intended for transplantation have been reported in previous studies to originate from forensic cases. An even higher portion (27%) when considering all purposes (Malm et al. 2016). Classifying a case as forensic does not preclude tissue donation or contact with next-of-kin. Clarifying the feasibility and appropriateness of such donations is however essential to ultimately expand the pool of available high-quality tissues. The Swedish National Board of Forensic Medicine (SNBFM) is committed to promoting tissue and organ donation and, in suitable cases, procure tissues from deceased individuals (Malm et al. 2016). The Autopsy Act (1995:832) also specifies the conditions under which such procedures may be permitted. According to the act, interventions on the body for purposes other than the forensic investigation (e.g., donation) are prohibited if they could compromise the results of the forensic examination. In Sweden, a forensic donation assessment is only initiated when the forensic unit is notified of a recently deceased individual, the police have requested a postmortem examination, and no obvious contraindications are present. Cases under investigation for homicide are rarely eligible for tissue donation; however, cases in which the manner of death is suspected to be suicide, accident or natural at that point may be considered.

Contact with next-ofkin is systematically undertaken in forensic cases irrespective of prior registration of yes or there is no registration in the national donor registry; however, no contact is initiated when the prior registration indicates a negative donation decision. The contacts are mainly made by phone and carried out by a range of personnel, including forensic professionals, but are predominantly conducted by staff at tissue establishments in Sweden. Following a positive response from the next-of-kin, the procurement of the tissue/tissues is performed either before, during or after the autopsy. A no response from next-of-kin is considered a negative response and no procurement is performed.

This study examines how next-of-kin interpretations of the deceased’s presumed wish to donate are influenced by manner of death, as well as by the deceased’s age and sex.

## Materials and methods

All donation assessments performed between 2018 and 2024 at the SNBFM were collected from the forensic medicine donation registry database and screened for eligibility and contraindication. Variables extracted include demographics, consent outcome, tissue types, manner of death, and national donation registry status. Descriptive statistics were used to present the population. Logistic regression models were fitted to assess the association between next-of-kin consent and manner of death, sex, and age group as covariates. Furthermore, odds ratios (ORs) were extracted since they provide an intuitive and standardized measure of effect size, quantifying how the odds of the outcome change between categories (for categorical variables). The use of ORs is particularly appropriate in this context, as they are the natural effect measure derived from logistic regression, and allow straightforward interpretation of associations. All variables were included based on the study objectives and were mutually adjusted in the model to fit the aim of the study. The interaction between sex and age groups were evaluated by comparing nested models using likelihood ratio tests. Although the interaction did not significantly improve overall model fit (*p*-value = 0.2031), it was retained in the final model to allow estimation of age-specific sex differences, in line with the specified research aims.

Multicollinearity was assessed using generalized variance inflation factors (GVIF) due to all covariates being indicator variables. Since there is an interaction term in the model, some covariates exceeded the $\sqrt{5}$ threshold. This was adjusted by transforming the indicator variables to sums instead of dummies which brought all the variables to GVIF < $\sqrt{5}$.

The study includes all eligible cases within the study period and is therefore based on the full study population rather than a sample, and consequently, the estimated ORs describe the true relationships within this population. The 95% confidence intervals (CI) are presented to reflect the precision of these estimates and the corresponding *p*-values are reported to describe the magnitude of the observed associations. The CI and *p*-values can also be used for future meta-analyses or Bayesian frameworks. Subgroup analyses were pre-specified and limited in numbers as part of the study objectives and no adjustment for multiple comparisons was applied, and as stated, *p*-values are used to describe the strength of the observed associations (Rothman 1990). All statistical analysis was performed using R (version 4.5.2) with the *emmeans* and *car* package to extract ORs and calculate GVIF respectively (R Core Team 2025; Fox 2019; Lenth and emmeans 2026).

## Results

### Selection and demography

**Fig. 1:**
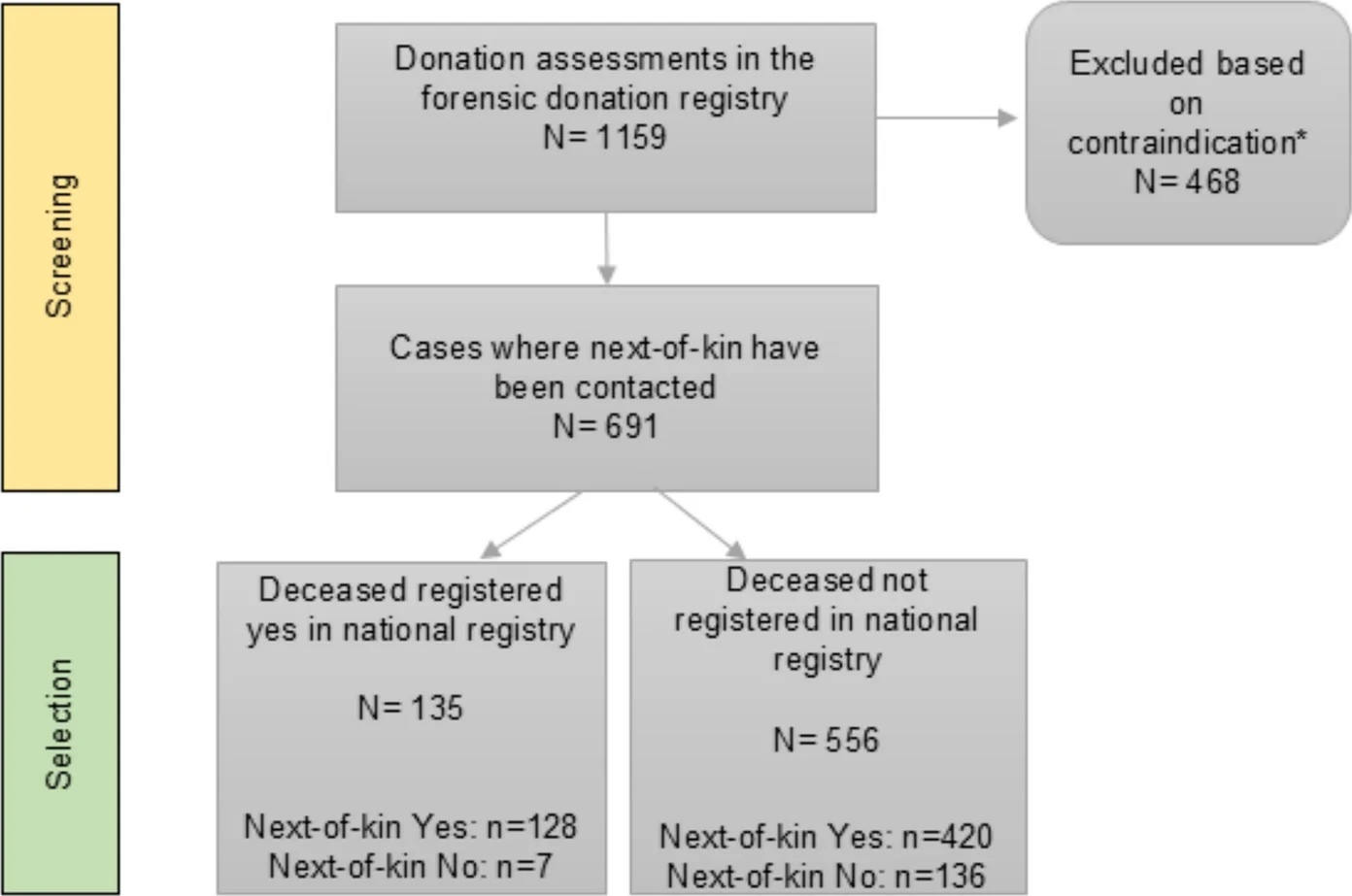
Flowchart of the screening and selection process from the national forensic donation registry. ^*^Reasons for exclusion included discovery of medical contraindications or risk behaviors, forensic considerations that rendered tissue donation inappropriate, or failure to obtain the mandatory blood sample within the required 24-h time frame, registration of a negative preference in the national donation registry or the absence of identifiable or contactable relatives

A total of 1 159 donation assessments were collected in the forensic medicine donation registry. Of these, 468 were discontinued and next-of-kin were not contacted. Donation assessments were discontinued for several reasons. The most common causes were the discovery of medical contraindications or risk behaviors, forensic considerations that rendered tissue donation inappropriate, or failure to obtain the mandatory blood sample within the required 24-h time frame (European Parliament of the Council 2004). Additional reasons included registration of a negative preference in the national donation registry and the absence of identifiable or contactable next-of-kin. In the remaining 691 cases, next-of-kin were approached, asked to interpret the deceased’s wishes, and they then either provided or declined consent for tissue donation. In some instances, next-of-kin did not respond, which was interpreted as a refusal. Of the 691 individuals, 135 had previously registered consent for donation in the national donation registry, whereas the remaining 556 had not (Fig. 1). The tissues recovered from the 548 individuals where consent was obtained from next-of-kin (128 with prior positive registration and 420 without prior registration) included heart valves, corneas, skin, auditory ossicles, temporal bone and meniscus (Table 1) and were intended either for transplantation or other medical purposes, including education or research.

**Table 1:** Performed procurements between 2018–2024 at the Swedish department of forensic medicine

Tissue	Performed procurements (n)*	Tissue retreived for
Heart valves	181	Transplantation
Corneas	406	Transplantation
Auditory ossicles	12	Transplantation
Temporal bone	163	Other medical purpose
Skin	105	Transplantation
Meniscus	162	Other medical purpose

*Each performed procurement can generate several tissues depending on tissue

**Fig. 2:**
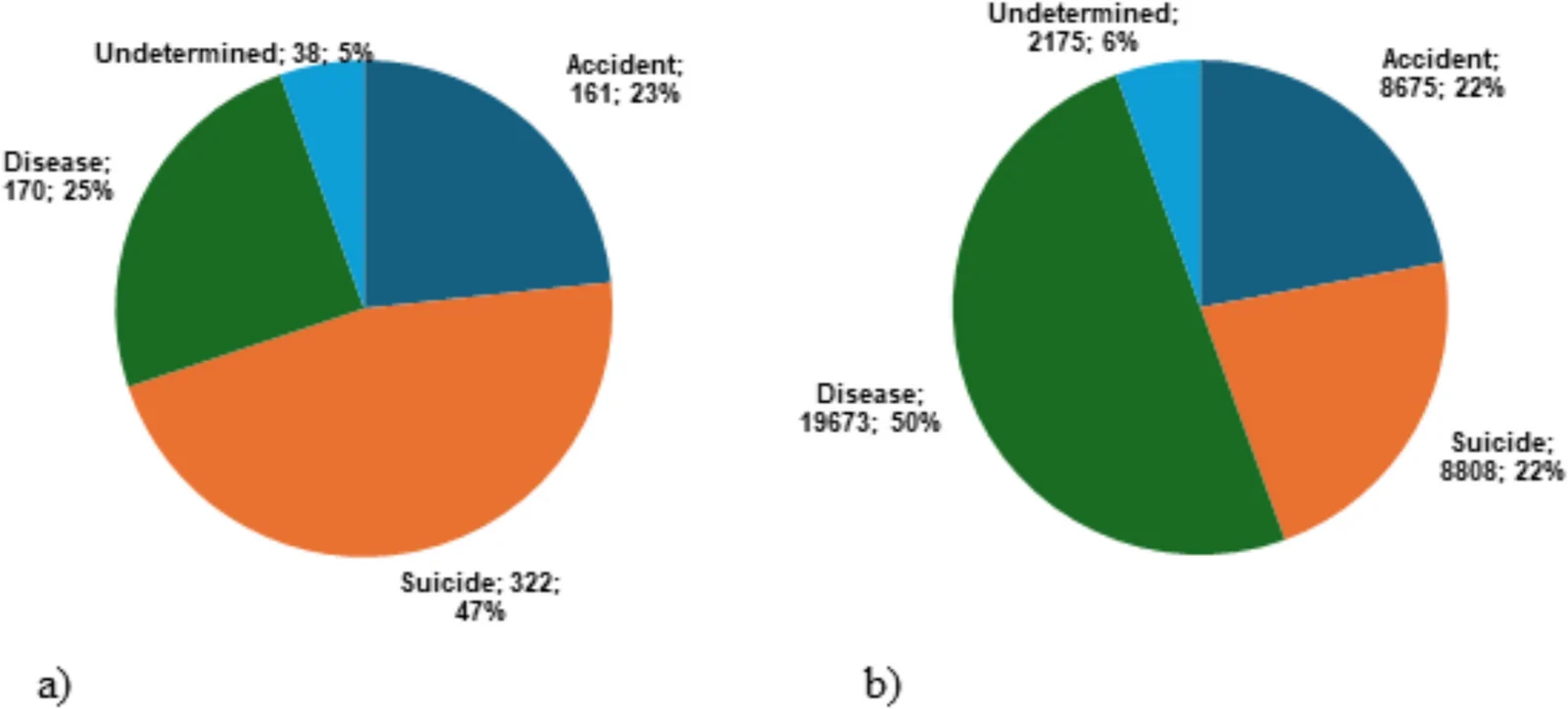
Number of individuals in each category for manner of death together with percentage of **a** all cases where next-of-kin have been contacted (2018–2024) for the possible donor population and **b** of the forensic autopsy population in Sweden during the same period (excluding cases under homicide investigation)

**Fig. 3:**
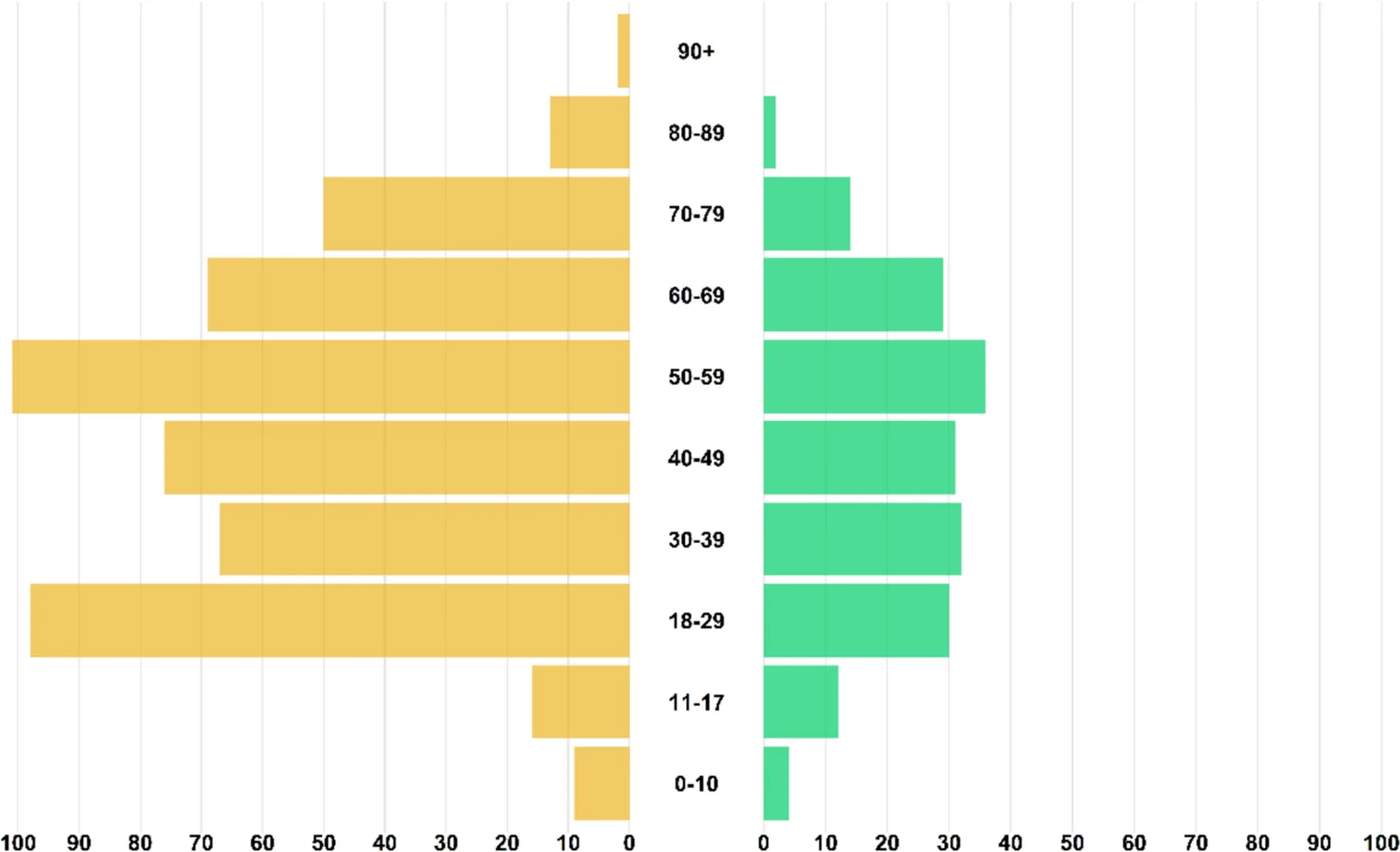
Frequency based on age groups for males (yellow) and females (green) where next-of-kin have been contacted (N = 691)

In the selected population (N = 691), the most common manner of death, irrespective of age or sex, was suicide (47%), followed by disease (25%), accident (23%), and undetermined (5%) for the period 2018–2024 (Fig. 2a). As a comparison, the most common manner of death in the total forensic autopsy population during the same period (excluding cases under homicide investigation) (N = 39 331), was disease (50%), followed by suicide (22%), accident (22%), and undetermined (6%) (Fig. 2b). The donor population comprised a higher proportion of men than women, reflecting the general demographic pattern observed in forensic death investigations. For both sexes, the largest age group was 50–59 years. The second largest age group was 18–29 years among men, whereas among women the distribution was relatively even across ages 18 to 69 years, with a slight predominance in the 50–59-year group (Fig. 3).

### Odds ratio

In order to assess differences in the likelihood of next-of-kin supporting donation according to the estimated manner of death, sex and/or age groups of the deceased, odds ratios were calculated using the subset of the population not registered in the national registry (N = 556). We found that next-of-kin were less likely to interpret the deceased’s wishes as supportive of donation when the manner of death was suicide or accident compared with disease (Table 2). Next-of-kin were 2.42 times more likely to interpret the deceased's wishes as positive in cases of disease than accident and 1.73 times more likely in cases of disease than of suicide. No major difference was observed between disease and undetermined manner of death. When including all age groups, no differences were observed in next-of-kin’s interpretation of the deceased’s wishes regarding donation based on the deceased’s sex (Table 2.) Depending on the variable of interest for likelihood for consent, the ORs is presented with both sexes or separated.

**Table 2:** Odds ratio with 95% confidence interval for the probability of relatives providing consent when no registered opinion was available

OR based on manner of death with disease as baseline			
Manner of death	Odds ratio (95% CI)	Inv. Odds ratio (95% CI)	*P*-value
Suicide	0.58 (0.33–1.02)	1.73 (0.98–3.04)	0.058
Accident	0.41 (0.23–0.75)	2.42 (1.34–4.36)	0.003
Unknown	0.62 (0.22–1.73)	1.62 (0.58–4.53)	0.359

OR based on sex with female as baseline			
Variable	Odds ratio (95% CI)	Inv. Odds ratio (95% CI)	*P*-value
Sex	1.13 (0.69–1.86)	0.88 (0.54–1.45)	0.621

Age group (years)	Odds ratio (95% CI)	Inv Odds ratio (95% CI)	*P*-value
*OR based on male age groups with 0–17 years as baseline*			
18–29	0.40 (0.09–1.89)	2.47 (0.53–11.53)	0.250
30–39	0.19 (0.04–0.92)	5.14 (1.08–24.40)	0.039
40–49	0.28 (0.06–1.33)	3.61 (0.75–17.34)	0.110
50–59	0.32 (0.07–1.49)	3.15 (0.67–14.79)	0.145
60–69	0.27 (0.06–1.28)	3.76 (0.78–18.17)	0.099
70–79	0.45 (0.08–2.39)	2.22 (0.42–11.83)	0.348
80 +	0.16 (0.02–1.05)	6.43 (0.95–43.35)	0.056
*OR based on female age groups with 0–17 years as baseline*			
18–29	2.69 (0.65–11.07)	0.37 (0.09–1.53)	0.171
30–39	0.86 (0.23–3.20)	1.16 (0.31–4.28)	0.827
40–49	1.77 (0.38–8.26)	0.56 (0.12–2.62)	0.465
50–59	4.46 (0.91–21.83)	0.22 (0.05–1.10)	0.065
60–69	4.48 (0.92–21.78)	0.22 (0.05–1.09)	0.063
70–79	1.65 (0.34–7.94)	0.60 (0.13–2.90)	0.530
80 +	0.68 (0.03–13.88)	1.48 (0.07–30.31)	0.800
*OR based on male age groups with corresponding female age groups as baseline*			
0–17	7.59 (1.30–44.29)	0.13 (0.02–0.77)	0.024
18–29	1.14 (0.37–3.52)	0.87 (0.28–2.69)	0.814
30–39	1.71 (0.62–4.71)	0.59 (0.21–1.62)	0.301
40–49	1.19 (0.32–4.42)	0.84 (0.23–3.14)	0.798
50–59	0.54 (0.14–2.08)	1.85 (0.48–7.11)	0.370
60–69	0.45 (0.12–1.76)	2.22 (0.57–8.68)	0.252
70–79	2.06 (0.48–8.82)	0.48 (0.11–2.07)	0.328
80 +	1.75 (0.08–39.17)	0.57 (0.03–12.85)	0.726

In the next step, ORs were examined separately for males and females. To assess differences in the likelihood of next-of-kin supporting donation among males not registered in the national registry (N = 424), ORs were calculated by age group. A notable finding was observed in the 30–39-year age group, where next-of-kin were 5.14 times less likely to interpret the deceased’s wishes as positive compared with the 0–17-year age group. No marked differences were observed in the other age groups compared to either the 0–17-year age group (Table 2). In the similar calculation for women (N = 132), a result was observed in the 50–59-and in the 60–69 year age groups, where next-of-kin were approximately 4.5 times more likely to interpret the deceased’s wishes as positive compared with the 0–17-year age group (Table 2). No marked differences were observed in the other age groups for women compared to the 0–17-year age group (Table 2).

As the last step, ORs were calculated according to sex and age group (N = 556). Next-of-kin were 7.59 times (CI 1.30–44.29) more likely to interpret the deceased’s wishes as positive in males (N = 24) aged 0–17-years (children) compared with females (N = 16) (children) of the same age group (Table 2). No marked differences were observed in the other age groups.

## Discussion

Post-mortem tissue donation from forensic autopsy cases provides a valuable source of suitable tissues, frequently obtained from younger and relatively healthy individuals. Engaging in dialogue with families regarding tissue donation remains a major challenge (Mackey-Bojack et al. 2007), particularly in cases involving children and adolescents who have died unexpectedly. However, findings from focus group studies suggest that parents perceive conversations about autopsy, organ donation, and research donation, although emotionally difficult, as potentially meaningful experiences (Crouch et al. 2023). Such discussions were found not only to support parents in their grieving process but also help them find additional meaning in their child’s life and death (Crouch et al. 2023). Most next-of-kin, interviewed within a year of the donor’s death, appreciated receiving a phone call to discuss consent as shown in a study by Wulff et al. (2013).

This study provides a comprehensive overview of the patterns and determinants of tissue donation authorization among cases referred to forensic medicine for medicolegal death investigations in Sweden. The results reveal that demographic factors and circumstances of death collectively influence the likelihood of next-of-kin decisions upon contact. While the manner of death is not yet fully established at this early stage following death, next-of-kin usually know some of the surrounding circumstances. It is reasonable to assume that relatives may have an idea about the manner of death and sometimes it is even obvious. Interestingly, previous studies, although focused on organ donation, have reported differences in consent related to the manner of death, albeit with somewhat conflicting results. These discrepancies may reflect differences in study design, population characteristics, and healthcare systems, as well as how and when next of kin are approached in each context. A key strength of the present study is its complete national coverage of all cases of unnatural deaths. In Sweden, responsibility for these death investigations is centralized to a single authority, ensuring complete case ascertainment and minimizing selection bias. However, contact with next of kin regarding tissue donation is not centrally coordinated and may vary in practice across tissue establishments. This study is based on a unique forensic dataset, and shows that various factors associated with each death and the deceased, influence the outcomes of postmortem donation processes.

The observation that a large proportion of donation assessments (40.4%) were discontinued highlights that there is a rigorous screening to identify suitable donors in a forensic setting. Common reasons for discontinuation were medical issues or forensic contraindications, where proceeding with donation could compromise the autopsy result. Other reasons include lack of consent, unavailable blood samples within the mandated time window or unknown time of death. All of these are covered by legal restrictions and represent factors that conform to international safety and governance standards for tissue donation (WHO Guiding Principles on Human Cell 2010; HealthCare EDftQoM 2021). A high refusal rate for tissue donation in general (not specifically forensic cases) is recognized as an important bottleneck for postmortem donation as discussed in a study by van Leiden et al. (2021). However, a nationwide approach in the Netherlands, including e-learning programs to enhance donor recognition, has led to a sufficient number of tissue donors to cover the national needs (Leiden et al. 2021).

The results of this study indicate that manner of death is an important predictor for consent. Comparing the distribution of manner of death between the potential forensic tissue-donor population where next-of-kin have been contacted between 2018 and 2024 (N = 691), with the distribution in the total forensic autopsy population in Sweden (excluding cases under homicide investigation) for the same period (N = 39 331), indicated some differences. It was approximately twice the proportion of suicides (47% vs. 22%) and half the proportion of deaths due to disease (25% vs. 50%) in the forensic tissue-donor population. The proportion of accidental deaths was however similar (23% vs. 22%).

Analysis revealed that families were less likely to provide consent when the death was due to suicide or accident compared with disease. These results suggest that next-of-kin might be hesitant toward tissue donation in cases of traumatic death, potentially due to emotional distress, societal perceptions of preventable deaths, or uncertainty regarding the deceased’s wishes (Ralph et al. 2014).

Sudden and traumatic deaths are accompanied by heightened emotional distress and may also include a portion of struggle of having to accept the death in combination with an, often unexpected, question about tissue donation, where relatives may lack knowledge about the subject and the deceased's views (Ralph et al. 2014). Conversely, disease-related deaths may theoretically allow for greater preparedness and alignment with medical contexts, increasing both awareness and acceptance of tissue donation. However, family members may find themselves to be in a challenging and vulnerable situation due to the loss regardless if it is disease-related or not (Latifi et al. 2025). It is known that an informed decision regarding donation is facilitated by a good contact between next-of-kin and the donor coordinator, and also adequate time to make the decision (Wulff et al. 2009). Additionally, research has shown that lack of information or insight in the donation process (eg. fear that the body of the deceased would be mutilated), may constrain next-of-kin from consenting (Siminoff et al. 2013). A contact with a physician of the forensic institute has been suggested to help families during the mourning phase to make an informed decision (Wulff et al. 2012).

Age specific analyses highlighted nuanced patterns in next-of-kin consent. In men, there was a considerably lower ORs of a positive interpretation of donation in the 30–39-year compared to other age groups. In contrast, the higher likelihood of positive interpretation for female cases aged 60–69 years may reflect communication of donation wishes with children as relatives rather than a grieving spouse.

In cases where the deceased was a child, the sex of the deceased was associated to the next-of-kin decision. Parents or guardians of boys were markedly more likely to respond favourably, compared to parents or guardians of girls. Speculatively, next-of-kin may be more inclined to find donation as acceptable for boys, while feeling a stronger need to protect the bodily integrity of girls, reflecting gender stereotypes and protective instincts. Differing interpretation based on sex has also been noticed in an earlier study regarding organ donation and family-decision making (Martinez et al. 2001). Those findings are in the context of adults and organ donation and many questions remain to explain the observed sex differences. Our study indicates differing patterns of parental interpretation in decision-making after a child’s death that underscore the necessity of tailored family communication strategies that consider both demographic and contextual sensitivities. However, these conclusions are tentative and needs to be confirmed and further explored in other contexts.

Finally, this study reinforces that successful facilitation of tissue donation is possible within forensic settings. Expanding education on tissue donation and promoting public’s trust in death-related institutions as well as public donation registration could substantially increase consent rates, particularly for unexpected deaths.

## Conclusion

The study highlights several practical implications for forensic tissue donation programs. The study clarifies that tissue donation is feasible and functionally appropriate even in cases of death undergoing investigation as unnatural. Both the manner of death as well as age and sex of the deceased influence how next-of-kin interpret the deceased’s wish to donate in forensic cases. Additionally, perceptions of donation among families of deceased children appear to differ by the child’s sex, highlighting the importance of tailored communication strategies.

## Limitations

Several limitations should be acknowledged. The study is restricted to cases assessed within a forensic medicine context, which may not generalize to hospital-based donation populations. Additionally, next-of-kin interpretation of the deceased’s wishes may be subject to recall bias or emotional influence, particularly in cases of sudden or traumatic death. In addition, the manner of death is not yet fully established at the point where next-of-kin are contacted, which may contribute to a source of error in conclusions regarding ORs for manner of death. No cross-referencing with other databases was performed to capture characteristics of relatives such as educational level, religious affiliation, or ethnicity (as potential confounding factors), nor to obtain information on how contact with relatives was conducted in cases managed by the tissue establishments. The study is limited to the analysis of data available within the forensic authority’s dataset. Lastly, the sample size for certain sex- and age-specific subgroups was small, potentially limiting certainty for some comparisons.

## Data Availability

The data are not publicly available due to privacy or ethical restrictions.

## Abbreviations

OR: Odds ratio
SNBFM: The Swedish National Board of Forensic Medicine
